# Myxoma Virus Infection Promotes NK Lysis of Malignant Gliomas *In Vitro* and *In Vivo*

**DOI:** 10.1371/journal.pone.0066825

**Published:** 2013-06-10

**Authors:** Henry Ogbomo, Franz J. Zemp, Xueqing Lun, Jiqing Zhang, Danuta Stack, Masmudur M. Rahman, Grant Mcfadden, Christopher H. Mody, Peter A. Forsyth

**Affiliations:** 1 Departments of Oncology, Biochemistry and Molecular Biology, University of Calgary, Calgary, Alberta, Canada; 2 Departments of Microbiology and Infectious Diseases, and Internal Medicine, University of Calgary, Calgary, Alberta, Canada; 3 Department of Molecular Genetics and Microbiology, University of Florida College of Medicine, Gainesville, Florida, United States of America; 4 Department of NeuroOncology, Moffitt Cancer Center and University of Southern Florida, Tampa, Florida, United States of America; University of Chicago, United States of America

## Abstract

Myxoma virus (MYXV) is a well-established oncolytic agent against different types of tumors. MYXV is also known for its immunomodulatory properties in down-regulating major histocompatibility complex (MHC) I surface expression (via the M153R gene product, a viral E3-ubiquitin ligase) and suppressing T cell killing of infected target cells. MHC I down-regulation, however, favors NK cell activation. Brain tumors including gliomas are characterized by high MHC I expression with impaired NK activity. We thus hypothesized that MYXV infection of glioma cells will promote NK cell-mediated recognition and killing of gliomas. We infected human gliomas with MYXV and evaluated their susceptibility to NK cell-mediated cytotoxicity. MYXV enhanced NK cell-mediated killing of glioma cells (U87 cells, MYXV vs. Mock: 51.73% vs. 28.63%, P = .0001, t test; U251 cells, MYXV vs. Mock: 40.4% vs. 20.03%, P .0007, t test). Using MYXV M153R targeted knockout (designated vMyx-M153KO) to infect gliomas, we demonstrate that M153R was responsible for reduced expression of MHC I on gliomas and enhanced NK cell-mediated antiglioma activity (U87 cells, MYXV vs. vMyx-M153KO: 51.73% vs. 25.17%, P = .0002, t test; U251 cells, MYXV vs. vMyx-M153KO: 40.4% vs. 19.27, P = .0013, t test). Consequently, NK cell-mediated lysis of established human glioma tumors in CB-17 SCID mice was accelerated with improved mouse survival (log-rank P = .0072). These results demonstrate the potential for combining MYXV with NK cells to effectively kill malignant gliomas.

## Introduction

Malignant gliomas remain incurable despite aggressive multi-modality therapy. Patients with these tumors have a median survival of only 12 to 15 months [1]–[3]. New approaches including oncolytic virotherapy, that rely on the ability of certain viruses to selectively replicate in and kill tumor cells and at the same time stimulate an immune response to the virus-infected and uninfected tumors, are actively been investigated [4], [5]. However, such immune responses, particularly of natural killer (NK) cells, can lead to premature viral clearance and abruptly end the anticancer effect of the virus [6]. In contrast to being a barrier to an effective oncolytic virotherapy, NK cells immunotherapy can be promoted by oncolytic viruses (OVs). OVs promote NK cell activity against tumor targets by up-regulating [7], [8] or down-regulating [7] NK cell activating or inhibitory ligands respectively on the surfaces of infected tumor cells. Specifically, parvovirus H1-PV infection of pancreatic ductal adenocarcinoma (PDAC) cells up-regulated NK activating ligand CD155 and down-regulated NK inhibitory ligand MHC I, resulting in enhanced sensitivity of H1-PV infected PDAC cells to NK cell-mediated lysis [7]. Thus, combining both therapies for an effective cancer treatment would require strategies that circumvent limitations associated with using either one therapy or the other.

Combinatorial virotherapy and immunotherapy (using NK cells) of gliomas appears promising [9] because glioma cells express several ligands recognized by NK cell receptors [10]–[17]. A major barrier to effective NK cell-mediated killing of malignant gliomas is high MHC I expression [18]. Therefore, down-regulation of MHC I expression on gliomas by OVs may improve their lysis by NK cells. In this setting, the OV infection of gliomas, even without viral replication, would down-regulate MHC I expression, followed by NK cell-mediated clearance of infected cells. This would eliminate the problem associated with premature viral clearance in oncolytic virotherapy since it is not necessary that the virus replicates in this case. An ideal OV should be relatively selective for tumor cells and nonpathogenic to humans. Myxoma virus (MYXV) has oncolytic activity against experimental gliomas [19] and down-regulates MHC I surface expression [20]–[22]. MYXV causes lethal myxomatosis in European rabbits but is nonpathogenic for all other vertebrate species including humans and mice [23].

In this study, we determined whether MYXV can enhance NK cell-mediated killing of gliomas *in vitro* and *in vivo* by reducing MHC I expression. We show for the first time that MYXV enhances NK cell-mediated cytotoxicity of glioma cells and accelerates clearance of established glioma tumors with significantly improved survival benefit *in vivo*, by down-regulation of MHC I expression.

## Materials and Methods

### Cell Lines

U87, U251, and U118 glioma cell lines were purchased from the American Type Culture Collection (ATCC; Manassas, VA). U87 glioma cell line was genetically engineered to express the firefly luciferase gene as previously described [24]. Cells were grown in DMEM/F12 (Life Technologies, Burlington, Canada) containing 10% fetal bovine serum at 37°C in a humidified 5% CO_2_ incubator. RT-PCR was used to routinely test cell lines for *Mycoplasma* contamination.

### Virus

The Lausanne strain derivative of Myxoma virus [25], designated MYXV, and the MYXV construct with the M153R gene deleted [26], designated vMyx-M153KO, were used in this study. Both viruses contain a green fluorescent protein (GFP) under the control of a poxvirus synthetic early-late promoter insert: for MYXV the GFP cassette is located between the open reading frames M135R and M136R of the MYXV genome while for vMyx-M153KO it is used to replace the M153R gene. Virus was propagated and titrated by focus formation on BGMK cells as described previously [27]. Dead virus (DV) was prepared by irradiating MYXV with UV light for 4 h.

### Cell Infection

For all studies, cells were infected with MYXV or vMyx-M153KO, or DV at indicated multiplicity of infection (MOI) for 1 h at 37°C, after which virus was removed from culture, cells washed with phosphate buffered-saline (PBS) and cultured further for 18 h with fresh culture medium before use. For mock infection, PBS was used to treat cells.

### Cell Viability Assay

A total of 1×10^4^ U87 and U251 cells, cultured in a 96 well plate, were infected with MYXV at indicated MOI for 24 and 48 h. The viability of cells was determined using alamar blue (Life Technologies) according to manufacturer's instruction.

### Measurement of Cell Surface Ligands

For quantitative analysis of the surface expression of NK ligands, a two-color cytofluorometric analysis (BDLSRII, BD Biosciences, Franklin Lakes, NJ) was carried out. Cells were stained with phycoerythrin (PE)-conjugated mouse monoclonal anti-human MICA/B, ULBP1–2, poliovirus receptor (PVR)/CD155 (all from R&D Systems, Minneapolis, MN), HLA–E (eBioscience, San Diego, CA), HLA class 1 ABC, clone 9.B.99 (Abcam, Cambridge, UK), Isotype-matched control Abs (R&D Systems), unconjugated ULBP3 (R&D systems) and Nectin-2 (Abcam) monoclonal antibodies (mAbs). For unconjugated mAbs, cells were further stained with PE-conjugated goat anti mouse IgG second reagent (R&D Systems).

### NK Cells Preparation

Human peripheral blood mononuclear cells (PBMC) were isolated from the blood of healthy volunteers by Ficoll-Hypaque centrifugation and NK cells were separated according to manufacturer's protocol using the MACS NK cell isolation kit II (Miltenyi Biotec, Auburn, CA). The separated NK cells were treated with 10 ng/ml recombinant human IL-2 (R&D Systems) for 5 days. Flow cytometric analysis to determine purity of NK cells showed that more than 95% of the cells were CD56^+^CD3^−^.

### Cytotoxicity Assay

NK cells were tested for their cytolytic activity against indicated target cells using the “aCella-Tox” kit (Cell Technology, Mountain View, CA) that employs the coupled luminescent technology for the detection of cytotoxicity as previously described [28]. For mAb blocking, mock-infected cells, as well as MYXV and vMyx-M153KO-infected cells were pre-incubated with 20 µg/ml of anti-MHC I (W6/32, Abcam) or isotype-matched control Ab (Becton Dickinson, San Jose, CA) before co-culture with NK cells. Because NK cells also mediate antibody-dependent cellular cytotoxicity via Fc receptor present on their cell surfaces, it is possible that blocking infected cells with anti-MHC I would trigger NK cell-mediated cytotoxicity. To address this likelihood, NK cells were pre-incubated as previously described [29] with 50% human serum (this masks Fc receptor expressed on the surface of NK cells, thus preventing triggering of NK cell-mediated cytotoxicity via mAb binding) before use.

### Measurement of Granzyme B

A total of 2×10^4^ U87 or U251 cells, either mock- or MYXV-, or vMyx-M153KO-infected were co-cultured for 24 h with 8×10^4^ NK cells. U87 or U251 cells, either mock or virus-infected, were also blocked with either 20 µg/ml anti-MHC I or isotype-matched control and used for the co-culture experiment. NK cells alone or respective target cells were used as control. Supernatants were collected and tested for release of granzyme B. The amount of secreted Granzyme B was determined using the human granzyme B ELISA kit (Abcam) according to manufacturer's protocol.

### Western Blot Analysis

For *in vitro* studies, cells were infected at MOI 2 with indicated viruses for 18 h. For *in vivo* studies, luciferase-expressing U87 cells were implanted into one hemisphere of mice brain. Twenty days after tumor implantation, mice were injected intratumorally (i.t.) with 5×10^6^ foci forming unit (ffu) MYXV or vMyx-M153KO or DV for 48 h. Afterwards, mice were anesthetized, tumor was taken out, fast frozen in liquid nitrogen, broken and smashed into smaller pieces, and lysed. Cell lysates were subjected to SDS-PAGE before transfer to nitrocellulose membranes (Schleicher & Schuell, Keene, NH) using the Mini-Protean II system (Bio-Rad, Hercules, CA). After transfer, blots were blocked in tris-buffered saline blocking buffer containing 5% low-fat milk for 1 h at room temperature to saturate the non-specific protein-binding sites on the nitrocellulose membrane. Membranes were incubated overnight with mouse monoclonal HLA class 1 ABC, clone EMR8-5 (Abcam), anti-human Nectin-2 Ab (R&D Systems), rabbit polyclonal anti-M-T7 Ab (generated in our Lab), anti-Luciferase Ab (Promega, Madison, WI) or rabbit polyclonal β-actin Ab (Cell signaling Technology, Danvers, MA) diluted in blocking buffer at 4°C with gentle agitation. Following a 1 h incubation period with peroxidase-conjugated secondary Ab at room temperature visualization was performed using Western Lightning ECL (PerkinElmer, Woodbridge, Canada).

### Immunohistochemistry

As described below, immunocompromised mice were implanted intracranially with luciferase-expressing U87 cells. Fourteen days following tumor implantation, mice were treated i.t. with DV, MYXV, or vMyx-M153KO viruses. Forty-eight hours after virus treatment, brains of mice were obtained and fixed with paraformaldehyde. Paraffin embedded brain sections were stained overnight at 4°C with rabbit polyclonal anti-M-T7 Ab (diluted 1∶1000 in PBS containing 10% goat serum). Biotinylated anti-rabbit IgG (Vector Laboratories, Burlingame, CA) was used as a secondary Ab. Immunostaining was carried out using the ABC immunohistochemistry (IHC) kit (Vector Laboratories). Sections were mounted and viewed using a Carl Zeiss microscope (Axiovert 200 M) mounted with a Carl Zeiss digital camera (AxioCam MRc).

### Orthotopic Glioma Model in CB-17 SCID Mice

An orthotopic glioma animal model, established with luciferase-expressing human U87 cells, was used to study MYXV-mediated NK cell clearance of this malignancy. Stereotactic technique was used to implant U87 cells (1×10^5^ cells/mouse) in the right putamen of CB-17 SCID mice (female, 6–8 weeks old, Charles River Canada, Constant, Canada) as previously described [30]. Tumor growth was monitored after systemic injection of luciferin substrate using the Xenogen IVIS 200 system to record bioluminescent signal emitted from tumors. Data were analyzed based on total photon flux emission (photons/s) in the region of interest over the intracranial space [24]. Mice were randomly placed in groups as indicated and treated i.t. with MYXV or vMyx-M153KO or DV, and 48 h later with NK cells. An initial minimal loss in body weight was observed during the first 48 h after virus administration, but mice recovered quickly. Virus was well tolerated. Mice did not lose weight following treatment with NK cells and appearance was normal. For survival study, animals were monitored until they lost 20% of body weight or were having trouble ambulating, feeding, or grooming, and then were killed.

### Ethics Statement

This study was carried out in strict accordance with the recommendations in the Guide for the Care and Use of Laboratory Animals issued by Canadian Council on Animal Care. All procedures were reviewed and approved by the University of Calgary Animal Care Committee (Permit Number: M09103). All surgery was performed under ketamine and xylazine anesthesia, and all efforts were made to minimize suffering. Also, for studies involving human blood samples from volunteers, approval was obtained from the University of Calgary Office of Medical Bioethics (Ethic ID: 18511). Participants provided their written informed consent to participate in this study. The Ethics Committee approved this consent procedure. All experiments involving the use of animals and human blood samples were conducted in Calgary, Canada.

### Statistical Analysis

The student *t* test was used to compare between two treatment groups. Log-rank tests were used in Kaplan-Meier analyses, and hazard ratio (HR) with corresponding 95% confidence interval (CI) was calculated. *P* values of less than .05 were considered as statistically significant. All statistical analyses and calculations were done using GraphPad Prism 5 (GraphPad Software Inc, La Jolla, CA). Analyses were two-sided and error bars were 95% CI.

## Results

### MYXV enhances NK cell-mediated killing of glioma cells *in vitro*

**Figure 1:**
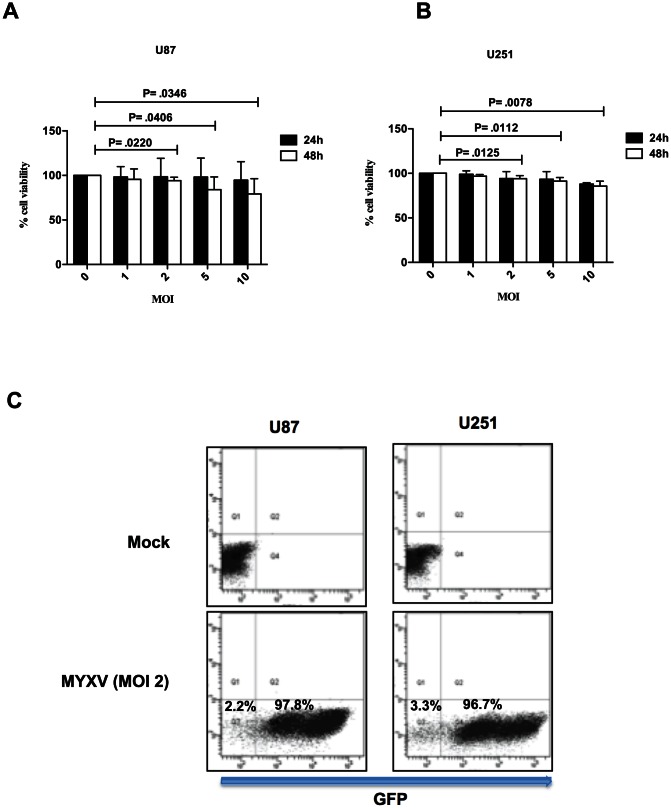
Viability and permissiveness of glioma cells to MYXV infection. Alamar blue cell viability assay was used to determine the percentage of viable U87 (**A**) and U251 (**B**) cells following 24 and 48 h infection with MYXV at the indicated MOI. Columns represent means of triplicate of one representative experiment. **Error bars** represent 95% confidence intervals, MOI =  multiplicity of infection. (**C**) Flow cytometric analysis to determine percentage of cells infected with MYXV. MYXV used contained GFP under the control of a poxvirus synthetic early-late promoter insert and located between the open reading frame M135R and M136R of the MYXV genome. GFP expression (detected on the FLI channel) indicates number of cells infected with MYXV (97.8% and 96.7% respectively for U87 and U251 cells). Mock-infected cells were used to set the gating. MYXV Infection was for 18 h. A representative of at least 5 independent experiments is shown. MYXV =  Myxoma virus, GFP =  green fluorescent protein.

To study MYXV-mediated antitumor activity of NK cells, we first determined the viability of glioma cells 24 and 48 h post infection with MYXV at MOI of 1, 2, 5, and 10. We observed that MYXV did not kill U87 cells alone by direct oncolysis within a 24 h time frame of infection (Figure 1A), even at an MOI of 10, while viability of U251 was only reduced at an MOI of 10, 24 h post infection (Figure 1B). At 48 h post infection, MYXV reduced the viability of both U87 and U251 cells at MOI 2, 5, and 10 (Figure 1A and 1B). Also, both U87 and U251 cell lines were highly permissive to MYXV infection with more than 96% of cells infected at a MOI of 2, 18 h post infection (Figure 1C, and [19]). On the basis of these data all subsequent *in vitro* experiments were performed using a MOI of 2 to infect glioma cells for 18 h.

**Figure 2:**
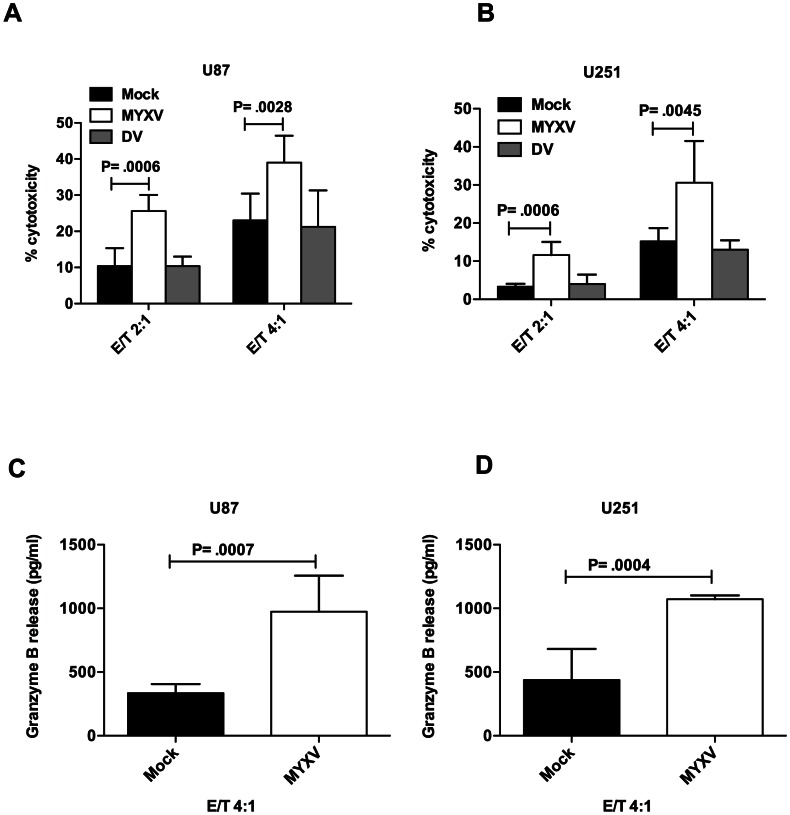
MYXV enhances NK cell-mediated killing of glioma cells. A 4 h cytotoxicity assay against 5000 U87 (**A**) or U251 (**B**) target cells, either mock or MYXV-infected was performed at indicated E/T ratios. Cells were infected at an MOI of 2 for 18 h before NK cell cytotoxicity was performed. Columns represent means of triplicate of one representative experiment. **Error bars** represent 95% confidence intervals, E/T =  effector (NK cells) to target (glioma cells) ratio, DV =  dead virus. Mock-infected and MYXV-infected U87 (**C**) and U251 (**D**) cells were co-cultured with NK cells for 4 h. Afterwards, supernatants were collected and analyzed by ELISA for granzyme B release. Baseline NK cells granzyme B release was 2020 pg/ml. The result shown is granzyme B release in NK/glioma co-culture minus baseline granzyme B release by NK cells alone. Columns represent means of triplicate of one representative experiment. **Error bars** represent 95% confidence intervals.

Next, the effect of oncolytic MYXV on antiglioma activity of NK cells was investigated. For this purpose U87 and U251 cells, either infected with MYXV or mock-infected, were co-cultured for 4 h with NK cells, after which the percentage NK cell cytotoxicity was determined. We observed that MYXV significantly enhanced NK cell-mediated lysis of both U87 (Figure 2A) and U251 cells (Figure 2B). Similar results were obtained using U118 glioma cell line (not shown). Also, MYXV significantly enhanced granzyme B release, a final mediator of NK cell-mediated cytotoxicity in NK/U87 co-cultures (Figure 2C) and in NK/U251 co-cultures (Figure 2D). The NK-sensitive cell line K562 was used as positive control and it showed 68% lysis by NK cells at E/T ratio of 4∶1 and 2072 pg/ml±151.5 granzyme B release in co-culture of NK/K562 (not shown).

### MYXV-enhanced NK cell-mediated clearance of U87 tumor results in prolonged survival

**Figure 3:**
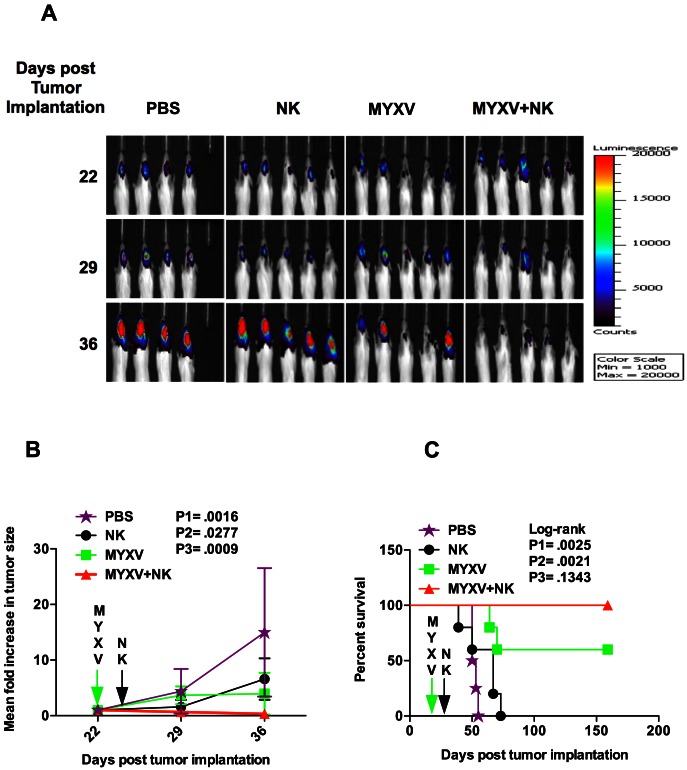
MYXV-enhanced NK cell-mediated clearance of U87 tumor results in prolonged survival. (**A**) Bioluminescence imaging (BLI) to monitor growth of orthotopic luciferase-expressing human U87 xenografts in CB-17 SCID mice. A total of 1.5×10^5^ IL-2-activated NK cells were administered intratumorally, 48 h (day 24 post tumor implantation) following intratumoral MYXV treatment (5×10^6^ ffu/mouse, day 22 post tumor implantation); PBS =  phosphate-buffered saline. A heat map indicating tumor size in 4 to 5 mice in each group is shown. (**B**) Luminescent intensities from U87 xenografts were quantified on day 0, 7 and 14 post-treatment (day 22, 29, and 36 post tumor implantation respectively) to monitor tumor clearance. Results show the mean fold-increase in tumor size after treatment (calculated as luminescent intensity on day 22/day 22, day 29/day 22, day 36/day 22 post tumor implantation). A statistically significant reduction in tumor size was observed in the MYXV+NK group compared with the other groups, 14 days post-treatment (36 days post tumor implantation, NK vs. MYXV+NK: 6.58±1.34 vs. 0.33±0.09 fold increase, difference = 6.25±1.34, 95% CI = 3.153 to 9.350, P1 = .0016; MYXV vs. MYXV+NK: 3.96±1.35 vs. 0.33±0.09 fold increase, difference = 3.63±1.35, 95% CI = 0.5121 to 6.750, P2 = .0277). Also, 7 days post-treatment (29 days post tumor implantation), a significant reduction was observed only in MYXV vs. MYXV+NK (3.66±0.58 vs. 0.66±0.07 fold increase, difference = 3.00±0.58, 95% CI = 1.663 to 4.344, P3 = .0009). **Error bars** represent 95% confidence intervals. (**C**) Survival curves showing that mice treated with MYXV+NK survived significantly longer than PBS (median survival for PBS-treated group vs. MYXV+NK was 51.5 days vs. 100%, hazard ratio (HR) = 30.89, 95% CI of HR = 3.333 to 286.2, log-rank P1 = .0025) or NK-treated mice (median survival for NK-treated group vs. MYXV+NK was 67 days vs. 100%, HR = 20.60, 95% CI of HR = 3.007 to 141.1, log-rank P2 = .0021) but not with MYXV-treated mice (median survival for MYXV vs. MYXV+NK-treated group was 60% vs. 100%, HR = 8.366, 95% CI of HR = 0.5188 to 134.9, log-rank P2 = .1343). Experiment was terminated on day 159 post tumor implantation.

The observed MYXV-enhanced antitumor activity of NK cells was tested *in vivo* in an orthotopic human U87 glioma model in immunocompromised mice. In order to simultaneously monitor tumor growth and mouse survival, luciferase-labeled U87 cells were used for the animal studies. The viability of luciferase-labeled U87 cells as well as their permissiveness to MYXV infection was similar to that of U87 cells (not shown). Combination i.t. treatment of established human U87-tumor with MYXV+NK dramatically reduced tumor size (Figure 3A). Quantified luminescent intensities showed tumor sizes, 7 and 14 days post-treatment, were reduced (1.5 and 3-fold, respectively) in mice treated with MYXV+NK; tumor sizes increased with either PBS (4.4 and 15-fold), or NK (1.6 and 6.6-fold) or MYXV (3.66 and 4-fold) monotherapy (Figure 3B). Mice treated with MYXV+NK survived significantly longer than PBS or NK-treated mice but not with MYXV-treated mice (Figure 3C). Median survival for PBS, NK, MYXV, or MYXV+NK-treated group was 51.5 days, 67 days, 60% of mice survived more than 159 days, or 100% of mice survived more than 159 days, respectively.

### MYXV down-regulates MHC I and Nectin-2

**Figure 4:**
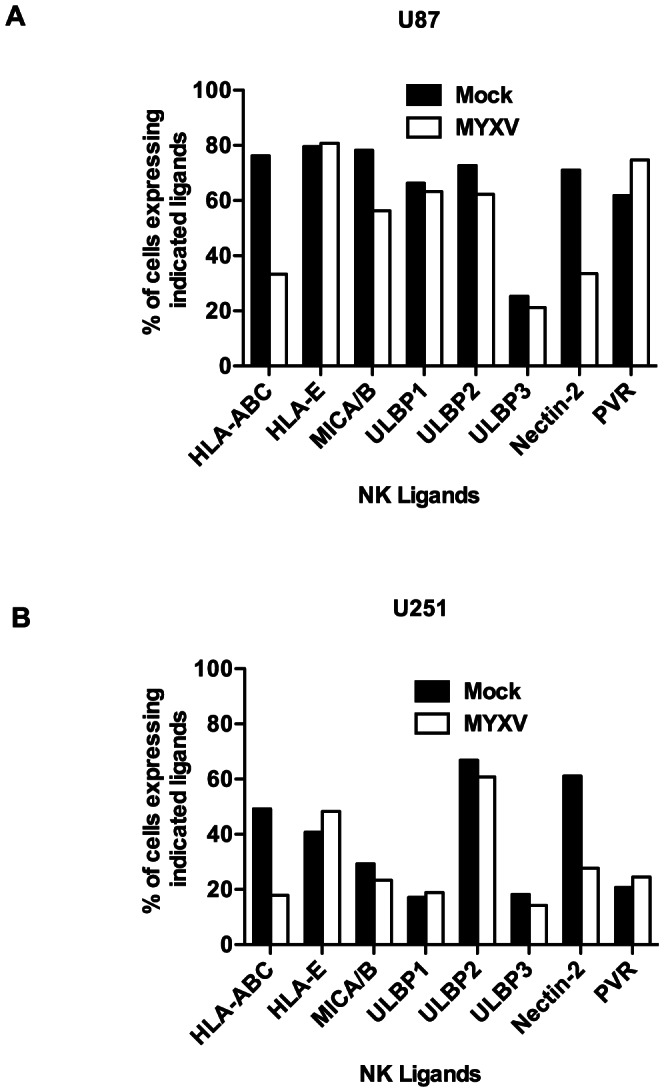
MYXV down-regulates MHC I and Nectin-2. Flow cytometric analysis for the expression of NK cell activating and inhibitory ligands in U87 (**A**) and U251 (**B**). One representative of at least 3 separate experiments is shown. MYXV =  Myxoma virus.

NK cell killing of its target involves signaling via its receptors that bind to target cells. It is possible that infection of glioma cells with MYXV modulates the surface expression of NK ligands expressed on gliomas and thereby influences NK cell-mediated cytotoxicity. We thus assessed the surface expression of a panel [HLA-ABC, HLA–E, MICA/B, ULBP1–3, Nectin-2, and poliovirus receptor (PVR)/CD155] of NK cell ligands by flow cytometry following MYXV infection (Figure 4A and 4B). We found that MYXV strongly (by more than 50%) down-regulated the surface expression of HLA-ABC and Nectin-2 in U87 and U251 cell lines (Figure 4A and 4B). Similarly, MYXV significantly down-regulated the surface expression of HLA-ABC and Nectin-2 in U118 cell line (not shown).

### MYXV enhances NK cell-mediated killing of glioma cells by MHC I down-regulation

**Figure 5:**
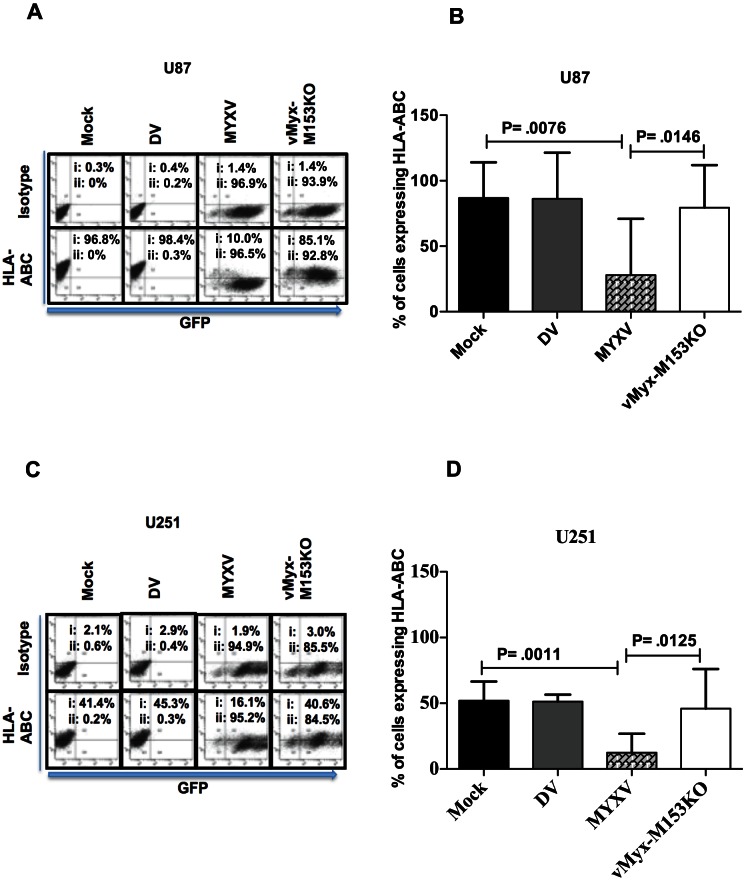
MYXV down-regulates MHC I via M153R. Dot plot showing the role of M153R in HLA-ABC down-regulation in U87 (**A**) and U251 (**C**) glioma cells and show percentage of cells infected with MYXV and vMyx-M153KO. HLA-ABC or isotype indicates number of cells stained with HLA-ABC or isotype Ab (detected on the FL2 channel; i =  percentage of cells with Ab staining); GFP indicates number of cells infected with MYXV or vMyx-M153KO (detected on the FLI channel; ii =  percentage of cells infected). Isotype control staining of mock-infected cells was used to set the gating. A representative of at least 3 independent experiments is shown. Flow cytometric analysis of U87 (**B**) and U251 (**D**) in triplicates and quantified. Columns represent means of triplicate of one representative experiment of two. **Error bars** represent 95% confidence intervals, DV =  dead virus, vMyx-M153KO =  mutated MYXV lacking M153R.

**Figure 6:**
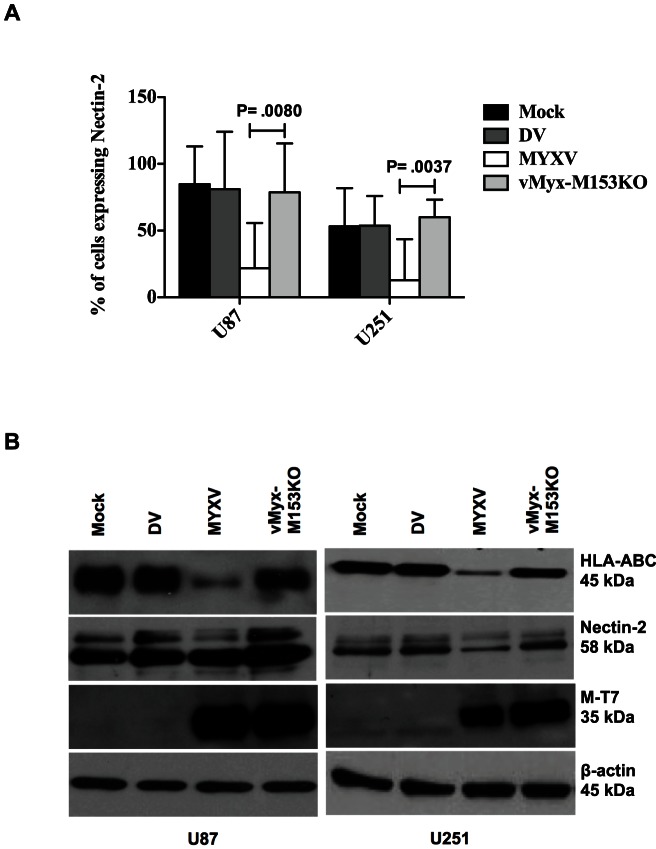
MYXV down-regulates Nectin-2 via M153R. (**A**) Flow cytometric analysis to determine the role of M153R in Nectin-2 down-regulation in glioma cells. Columns represent means of triplicate of one experiment. **Error bars** represent 95% confidence intervals. (**B**) Western Blot showing down-regulation of HLA-ABC and Nectin-2 by MYXV. DV =  dead virus control; M-T7, an early gene product of MYXV, shows MYXV and vMyx-M153KO infection while β-actin serves as loading control.

The results obtained so far suggest that MYXV inhibits expression of both NK co-activating and inhibitory ligands. HLA-ABC (MHC I) functions as an NK cell inhibitory ligand and reduced expression favors an activating signal. On the other hand, Nectin-2 functions as an NK cell activating ligand and reduced expression favors an inhibitory signal. The fact that NK cell-mediated killing of glioma cells was enhanced by MYXV, despite down-regulation of Nectin-2, suggests that MHC I down-regulation may be the mechanism of enhanced NK cell-mediated lysis. We therefore used a loss of function approach employing a targeted knockout mutant MYXV that has lost its ability to down-regulate MHC I. The M153R protein of MYXV is a virus-encoded E3 ubiquitin ligase that is expressed as early as 4 h after infection and independently of viral DNA replication, and down-regulates MHC I expression [21], [22], [26]. The deletion of MYXV's M153R gene restores MHC I expression. Using a mutated MYXV that has M153R gene deleted (designated vMyx-M153KO) to infect glioma cells, we found that the loss of M153R inhibited the ability of MYXV to down-regulate MHC I surface expression (Figure 5A–D) and total MHC I protein (Figure 6B) in U87 and U251 glioma cells. Also, M153R specifically mediated the cell surface down-regulation of Nectin-2 expression (Figure 6A) as well as total Nectin-2 protein (Figure 6B). Like MYXV, infection with vMyx-M153KO did not affect expression of other NK ligands including HLA–E, MICA/B, ULBP1–3, and PVR (not shown). Of note, comparable degrees of infection were found with both MYXV and vMyx-M153KO (Figure 5A and 5C) showing that the difference in MHC I expression was not an artifact of differences in infection.

**Figure 7:**
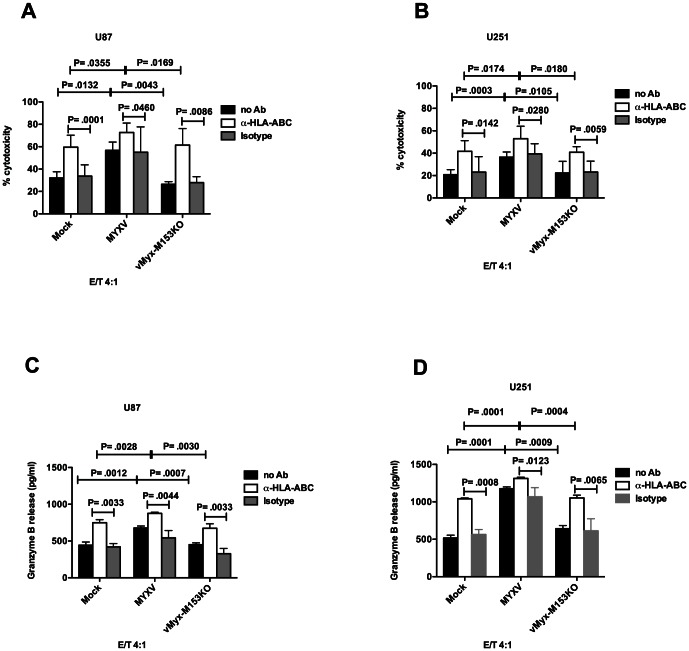
MYXV enhances NK cell-mediated killing of glioma cells via MHC I down-regulation. M153R-mediated down-regulation of MHC I or blockade of MHC I with mAb results in enhanced NK cell-mediated killing of U87 (**A**) or U251 (**B**) target cells. For mAb blocking, mock-infected or virus-infected cells were pre-incubated with 20 µg/ml MHC class I Ab (W6/32) or isotype-matched control Ab for 30 min before co-culture with NK cells. Columns represent means of triplicate of one representative experiment. **Error bars** represent 95% confidence intervals. Supernatant was collected from 4 h co-cultures of NK cells and Mock- or MYXV- or vMyx-M153KO-infected U87 (**C**) or U251 (**D**) target cells, that were pre-incubated with either 20 µg/ml MHC class I Ab (W6/32) or isotype-matched control, and analyzed by ELISA for granzyme B release. Baseline NK cells granzyme B release was 1624 pg/ml. The result shown is granzyme B release in NK/glioma co-culture minus baseline granzyme B release by NK cells alone. Columns represent means of triplicate of one representative experiment. **Error bars** represent 95% confidence intervals.

Next, we performed NK cell cytotoxicity experiments to see whether M153R mediates the MYXV-enhanced NK cell-mediated lysis of glioma cells (as seen in Figure 2). We observed that loss of M153R function abrogated MYXV-mediated enhanced NK cell-mediated lysis of U87 cells (Figure 7A) and U251 cells (Figure 7B). Also, blockade of MHC I expression on mock-infected and virus-infected U87 cells (Figure 7A) or U251 cells (Figure 7B) with mAb enhanced NK lysis. This suggests that MHC I down-regulation is sufficient to enhance NK cell-mediated lysis of glioma cells. Granzyme B secretion was also enhanced by down-regulation of MHC I either with MYXV or with mAb in NK/U87 co-cultures (Figure 7C) and NK/U251 co-cultures (Figure 7D). Taken together, the mechanism of MYXV-enhanced antiglioma activity of NK cells is M153R-mediated down-regulation of MHC I expression.

### MYXV down-regulates MHC I expression in established glioma tumors *in vivo*

**Figure 8:**
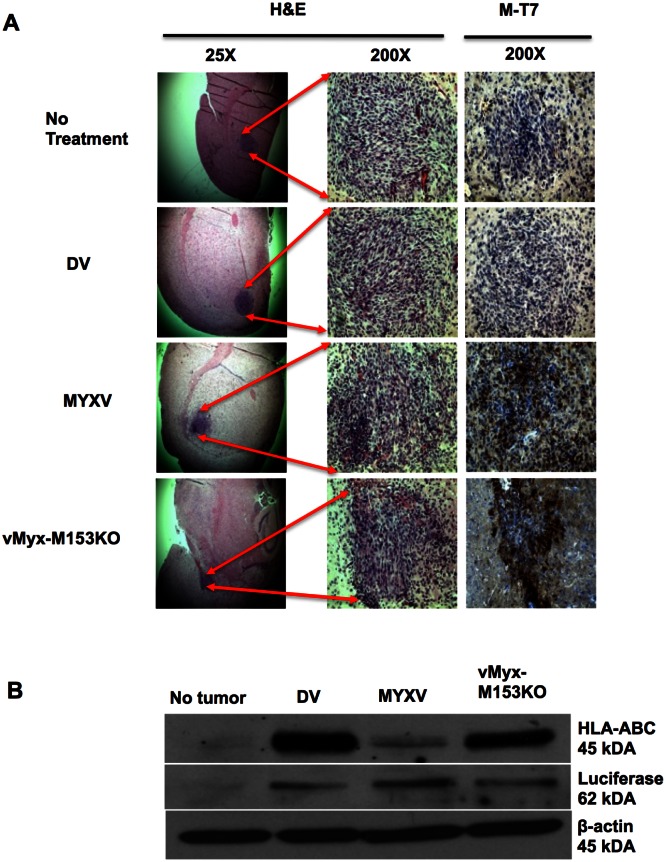
MYXV down-regulates MHC I in U87 *in vivo*. (**A**) Immunohistochemistry was performed on paraffin sections of mice brains with established U87 tumors that were treated with indicated viruses for 48 h. Hematoxylin and Eosin (H&E) stain shows tumor, with the arrows indicating tumor location in the brain. Brown M-T7 stain represents MYXV early protein staining within the tumor. Photomicrographs were magnified 25X and 200X. (**B**) Western Blot of protein extracts from the U87 tumor-bearing hemisphere of mice brains. As control for human HLA-ABC Ab specificity, the contralateral hemisphere without tumor was used. Luciferase expression shows tumor, β-actin serves as protein loading control.

To determine if MHC I down-regulation and enhanced glioma killing occurred *in vivo*, we first investigated if MYXV protein is expressed within glioma tumors and down-regulates MHC I expression *in vivo*. For this purpose, human glioma xenografts were inoculated into the brains of SCID mice and treated i.t. with MYXV or vMyx-M153KO. We then stained tumor sections for M-T7 (an early MYXV protein) expression and found it present in MYXV- and vMyx-M153KO-treated mice (Figure 8A). We then showed that MHC I expression was reduced *in vivo* by M153R expressing MYXV using Western blots (Figure 8B).

### M153R mediates enhanced NK cell-mediated clearance of glioma *in vivo*

**Figure 9:**
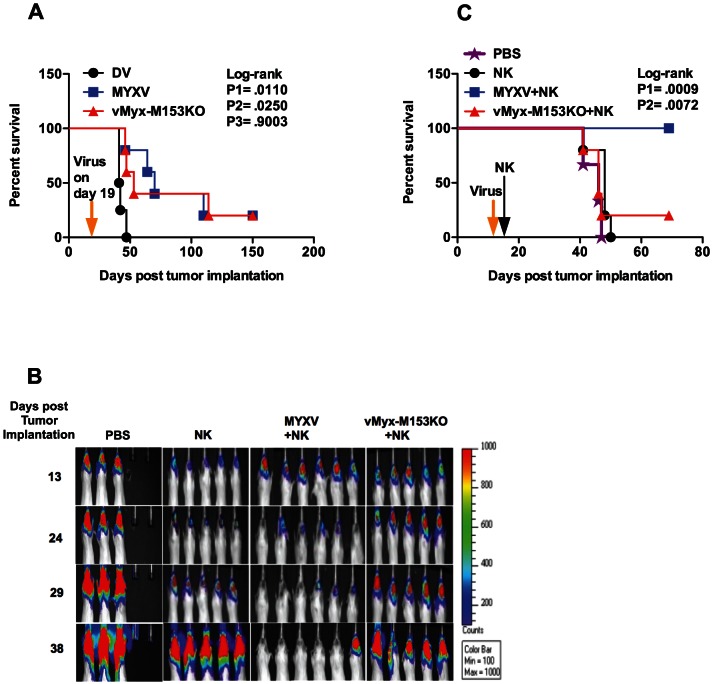
M153R mediates enhanced NK cell-mediated clearance of U87 tumor with prolonged survival. (**A**) Survival study to compare oncolytic capacity of MYXV with vMyx-M153KO. Dead virus (DV) was used as control. CB-17 SCID mice with established tumors were treated with either DV (n = 4) or MYXV (5×10^6^ ffu/ml, n = 5) or vMyx-M153KO (5×10^6^ ffu/ml, n = 5) on day 19 post tumor implantation. Log-rank P1 (.0110) represents MYXV vs. DV, log-rank P2 (.0250) represents vMyx-M153KO vs. DV, log-rank P3 (.9003) represents MYXV vs. vMyx-M153KO. (**B**) A heat map indicating tumor size in 5 to 6 mice in each group is shown. CB-17 SCID mice with established tumors were treated with either NK or a combination of MYXV+NK or vMyx-M153KO+NK. NK cells (1.5×10^5^ NK cells) were administered intratumorally, 48 h (day 16 post tumor implantation) following intratumoral MYXV (5×10^6^ ffu/ml) or vMyx-M153KO (5×10^6^ ffu/ml) treatment (day 14 post tumor implantation). Images show accelerated NK clearance in MYXV+NK-treated group compared with NK or vMyx-M153KO+NK-treated group. (**C**) Survival curves showing that mice treated with MYXV+NK survived longer than NK-treated mice (HR = 31.26, 95% CI of HR = 4.097 to 238.5, log-rank P1 = .0009) and vMyx-M153KO+NK-treated mice (HR = 0.05532, 95% CI of HR = 0.006701 to 0.4567, log-rank P2 = .0072).

Because viral oncolysis may contribute to tumor clearance (Figure 3), we investigated the oncolytic capacity of both MYXV and vMyx-M153KO *in vivo* by performing survival studies using U87 glioma model. We found that MYXV and vMyx-M153KO similarly prolong survival of U87-tumor bearing mice compared with dead virus control (Figure 9A). We next determined the effect of MHC I down-regulation on NK cell-mediated clearance of gliomas *in vivo*. We found a rapid clearance of tumors in MYXV+NK-treated group (Figure 9B). In contrast, the tumor size increased in NK or vMyx-M153KO+NK-treated group. An enhanced survival benefit was also observed in mice treated with MYXV+NK (median survival for vMyx-M153KO+NK-treated group was only 46 days while 100% of mice survived more than 69 days for MYXV+NK-treated group (Figure 9C).

## Discussion

The search for alternative treatment options for malignant gliomas continues to remain a priority, since conventional therapies are not curative. In this study, we combined two safe biotherapeutic approaches (virotherapy and immunotherapy) to treat malignant gliomas. This combination harnesses the nonpathogenicity of the virus to humans [23] as well as the MYXV's immunomodulatory property of down-regulating cell surface MHC I expression [20]–[22]. This suppresses signaling via NK inhibitory ligand and thus favors a NK activating response.

Previous work by others elegantly demonstrated that the PHD/LAP-domain protein M153R of MYXV down-regulates MHC I expression during infection in human melanoma cell lines, HeLa cells, and BGMK cells [21], [22], [26] and deletion of M153R from the virus genome restored MHC I expression. We similarly found that M153R down-regulates MHC I expression on glioma cells, which in turn leads to enhanced NK cell-mediated lysis of glioma cell.

Our *in vivo* findings (MYXV+NK group vs. vMyx-M153KO+NK group) validate the role of M153R in the MYXV-mediated enhanced NK cell-mediated lysis of glioma tumors *in situ*. We recognize that viral oncolysis contributes to tumor clearance and mouse survival. However, the oncolytic effect of MYXV (occurs between day 7 and day 14 following treatment, Figure 3A and 3B) begins much later than MHC I down-regulation (occurs within 48 h of MYXV treatment, Figure 8B) and early-enhanced rapid glioma clearance occurs within 5 days post NK treatment, Figure 3A and 3B). Hence, using the loss of function approach, we demonstrate that: 1) Like in other cell types, MYXV down-regulates MHC I expression in glioma cells *in vitro* and *in vivo* via M153R. 2) M153R-mediated down-regulation of MHC I expression is responsible for enhanced NK cell-mediated lysis of glioma cells *in vitro* and, at least in part, for enhanced NK cell-mediated lysis of established glioma tumors *in vivo*. 3) Resistance of glioma cells/tumors to NK cell-mediated lysis can be partly explained by the high MHC I expression.

In addition to the genetic approach we used the MHC I blocking antibody W6/32 mAb and found that blocking MHC I expression on the surface of glioma cells is sufficient to overcome resistance of glioma cells to NK cells, resulting in enhanced NK cell-mediated lysis of glioma cells. This corroborates findings by others demonstrating that mAb blocking of resistant LN229 glioma cells with W6/32 sensitizes glioma to NK cytolysis [18].

Recently, MHC I expression was described to define human NK cell activation/inhibition threshold [31]. Such a threshold allows small changes in the target cell surface phenotype to dramatically alter susceptibility to NK cells, and transition from a resistant target to a sensitive target occurs over a relatively narrow range of MHC I expression [31]. In this regard, our observations that combination therapy with MYXV+NK produced an early-enhanced rapid glioma clearance suggest that MYXV functions by lowering the inhibition threshold for NK cell activation.

Our findings also reveal M153R to be responsible for Nectin-2 down-regulation. Nectin-2, like PVR, binds to DNAM-1, a NK cell co-activating receptor involved in NK lysis of human glioblastoma [15]. Despite MYXV-mediated down-regulation of Nectin-2 we still saw enhanced NK cell-mediated killing of glioma cells. The reason for this may be partly due to the fact that DNAM-1 signals via two axes: DNAM-1/PVR and DNAM-1/Nectin-2. Since PVR expression was not affected by MYXV, it is possible that NK cells send activating signals through DNAM-1/PVR axis, which may compensate for the reduction in Nectin-2 expression.

We are aware of several limitations of our study. We chose to examine human gliomas, but this necessitated the use of immunocompromised mice for *in vivo* experiments. MYXV infection as monotherapy does prolong survival in immunocompromised animal models (Figure 3C; [19]) and survival is further enhanced from 60% to 100% in mice that received NK cells in addition to MYXV (Figure 3C). The importance of NK cell antiglioma activity in the context of MYXV infection in immunocompetent animals is unknown. Recent reports for oncolytic herpes simplex viral (oHSV) therapy *in vitro*, using human U87 and U251 cell lines, showed an enhanced IL-15-activated NK cell-mediated cytotoxicity against oHSV-infected cells compared to uninfected cells [6]. In both human U87 xenograft and mouse 4C8 syngeneic immunocompetent glioblastoma models, the authors demonstrated that depletion of NK cells increased oHSV titres and enhanced oncolytic efficacy of the virus [6]. The authors argue that although oHSV infection of experimental glioblastoma leads to rapid recruitment of NK cells with activated phenotype to the brain, the response did not facilitate antitumor effects but rather led to premature viral clearance, limiting oHSV anticancer effect. It is important to mention that the focus of their *in vivo* study was on viral oncolysis rather than a possible combinatorial oHSV and/or NK cell therapy. Based on the above, one can speculate that in immunocompetent models NK cells may prevent MYXV replication and limit viral oncolytic efficacy. In our model, staining of brain tissues from MYXV-treated U87 tumor-bearing mice (treatment was for 48 h) for CD49b expression (marker for endogenous mouse NK cells) was negative (not shown). Our approach proposes that infection of gliomas with MYXV with/without viral replication will significantly suppress MHC I expression and activate NK cells to clear the infected tumor. Some other agents including antibodies and cytotoxic drugs that down-regulate MHC I expression can be used in combination therapy with NK cells. However, because of toxicity and potential to induce resistance or enhance invasion of the tumor [32], [33], alternative approaches are needed. Our approach would require i.t. injection of MYXV followed by adoptive transfer of enriched and ex-vivo activated NK cells directly to tumor site in the brain. MYXV is safe in humans [34] and shows tropism for human tumors [19], [35]–[37]. Furthermore, the brain is considered to be immunologically privileged because most of its cells express very low or undetectable MHC I [38] and so the possibility of MYXV down-regulating MHC I on other brain cells is unlikely. This eliminates translational concern of specificity of MYXV to tumor and its effect on other brain tissues. One possible disadvantage of using MYXV in the clinics is that MYXV-mediated down-regulation of MHC I may render glioma cells more resistant to CD8+ T cell-mediated killing. This disadvantage is however not of concern here since we are not proposing the use of CD8+ T cells, but NK cells.

A clinical trial in patients with recurrent malignant glioma using autologous IL-2-activated-NK cells as monotherapy was safe but only partially effective [39], possibly due to local immunosuppression and MHC I expression [9]. Since both MYXV and NK cell administration are feasible in humans, it is possible that with MYXV+NK therapy, MYXV will lower the threshold of MHC I expression enough to allow a broad cytolytic effect by NK cells. Moreover, it is possible that the MYXV-augmented activity of NK cells might allow for approaches to reduce the toxicity of NK cell therapy. These studies provide the rationale for a clinical trial using MYXV+NK. A technical challenge would be how to administer NK cells i.t. to the same patient, days after performing a tumor resection, without having to perform another surgery. In a previous clinical study, a reservoir/catheter system was used to administer lymphokine-activated killer (LAK) cells to human glioma patients. Here, following resection, the catheter was placed into the postoperative tumor cavity through which LAK cells were administered over 5 days period [40]. This clinical study provides insight into how this challenge may be overcome.
